# A framework for predicting odor threshold values of perfumes by scientific machine learning and transfer learning

**DOI:** 10.1016/j.heliyon.2023.e20813

**Published:** 2023-10-10

**Authors:** Luis M.C. Oliveira, Vinícius V. Santana, Alírio E. Rodrigues, Ana M. Ribeiro, Idelfonso B. R. Nogueira

**Affiliations:** a LSRE-LCM - Laboratory of Separation and Reaction Engineering – Laboratory of Catalysis and Materials, Faculty of Engineering, University of Porto, Rua Dr. Roberto Frias, 4200-465, Porto, Portugal; b ALiCE - Associate Laboratory in Chemical Engineering, Faculty of Engineering, University of Porto, Rua Dr. Roberto Frias, 4200-465, Porto, Portugal; c Department of Chemical Engineering, Norwegian University of Science and Technology, Sem Sælandsvei 4, Kjemiblokk 5, Trondheim, Norway

**Keywords:** Transfer learning, Odor thresholds, Machine learning, Artificial neural networks, Graph convolutional networks, Feedforward neural networks

## Abstract

Knowledge of odor thresholds is very important for the perfume industry. Due to the difficulty associated with measuring odor thresholds, empirical models capable of estimating these values can be an invaluable contribution to the field. This work developed a framework based on scientific machine learning strategies. A transfer learning-based strategy was devised, where information from a graph convolutional network predicting semantic odor descriptors was used as input data for the feedforward neural network responsible for estimating odor thresholds for chemical substances based on their molecular structures. The predictive performance of this model was compared to a benchmark odor threshold prediction model based on molecular structures that did not utilize transfer learning. Furthermore, the prediction was compared to a correlation previously proposed in the literature and a dummy regressor. Results demonstrated that the transfer learning-based strategy displayed a better predictive performance, suggesting this technique can be useful for predicting odor thresholds.

## 1 Introduction

An odor, as sensed by a human individual, has two relevant properties: intensity (the strength of the smell) and character (what the smell is like). The way these properties are sensed is influenced by an individual's prior experiences and expectations. They determine the extent to which they can be affected by odorous air pollution [1].

Odor thresholds can be divided into two types: detection threshold (ODT), the level at which the human olfactory sense can detect a certain smell but not recognize it, and recognition threshold, the level at which that smell can be recognized [2]. Knowledge of thresholds is very important for the industry, not only in terms of process functioning (as in the perfume industry) but also in terms of safety, as many substances can have severe negative health effects at certain concentrations.

There are several methods for measuring odor thresholds. The most widely used type of methodology is the dilution-to-threshold methods [3]. Odor thresholds are difficult to measure, involving a complex process that requires the usage of human subjects. For this reason.

The dilution-to-threshold method involves presenting subjects with a number of odor samples mixed with odorless air, increasing odor concentration, starting with a sample below the detection threshold. Subjects are exposed to each level of dilution multiple times. In the subtype of dilution-to-threshold methods, forced-choice methods, trained subjects receive odorous samples along with clean samples and are instructed to identify the presence of an odor. The detection threshold will be the level at which a subject can distinguish the difference between the diluted odor sample and the odorless sample [4].

Olfactometry makes use of olfactometers, instruments wherein a human subject is able to detect ambient odors. Scentometry, a type of olfactometry, is a type of odor concentration evaluation that requires using a device called a Scentometer® - a box with several air inlets and two sniffing ports. At least two of the air inlets contain activated charcoal filters to remove odor and generate clean air. In contrast, the remaining inlets have different diameters, which are used to allow a range of dilutions of odorous air samples to be tested. Subjects start with the samples with the lowest odor concentration and successively test the increasingly higher concentration odorous samples. When the subject can detect the odor for the first time, the odor threshold is considered reached [4]. They function very similarly to olfactometers, the main difference being that with olfactometers, there is usually a human operator in charge of sample delivery, while the subjects inhale through a sniffing port to detect the presence of odor [4].

Practical measurement of odor thresholds can have some associated uncertainty, as it requires the usage of human subjects and subjective perceptions. Furthermore, to identify reliable threshold data can be an expensive procedure. Due to these limitations, researching empirical models with the ability to estimate odor thresholds for certain compounds can be an invaluable contribution to this field.

Other models for ODT prediction have been developed in the past. Rodríguez et al. (2011) [5] developed a correlation for predicting ODTs in air, based on the physical properties of the odorants. This model relied on simplifications that may have affected its predictive capability. The model is based on partition coefficients between the different phases of the considered theoretical model. Because of the unavailability of data for those coefficients, a certain number of simplifications have to be made so that the model can have practical use. For instance, the partition coefficient for the water was taken as the partition coefficient for the aqueous phase. These simplifications lead to severe limitations on the model, such as the inability to predict ODTs for odorants with no miscibility with water, and the lack of distinction between enantiomers. These types of simplifications are not necessary in empirical models such as the one envisioned in this work.

Hence, in this work, a framework to predict odor thresholds using an empirical model will be proposed. This framework will be based on scientific machine learning strategies, namely Graph Neural Networks, Feed Forward Neural Networks, and Transfer learning. For this purpose, two databases were built, one composed of several chemical compounds and their respective odor descriptor and another with the corresponding thresholds. As the literature lacks information regarding ODTs, this work uses Transfer Learning (TL) to develop the proposed models. Therefore, we transfer known and vastly available information to build a model for the ODT.

## 2 Methodology

The methodology proposed here is based on certain machine learning models' capacity to learn the correlation between a given chemical structure and a corresponding property of that compound. All the models machine learning models presented here were originally designed for this work. All the models machine learning models presented here were originally designed for this work.

Several works have been published in other fields demonstrating this. For example, as seen in Zeng et al. (2018) [6], who applied graph convolutional networks (GCN) to the prediction of polymer properties and Yang et al. (2019) [7], who built graph convolutional models for the prediction of molecular properties based on molecular graph structures. Ryu et al. (2019) [8] used Bayesian graph convolutional networks in molecular property prediction.

The core part of predicting a property from a molecular structure is the availability of data that provides this information. In the case of ODT, this information is very scarce. Hence, this work uses the transfer learning technique by developing two Artificial Neural Networks (ANNs).

The first ANN will be used to learn the relationship between a given molecule and its corresponding molecular descriptor, in this case, the odor descriptor. This is done for two main reasons. First, it is necessary to establish a procedure to convert molecular structures into numerical information. For this purpose, the graph neural network is well known. The other reason is the underlying assumption that there should be an unknown correlation between the ODT and the odor descriptor. As it is easier to find the descriptor information than the ODT, the proposed methodology uses transfer learning to leverage this hypothetical correlation.

A graph convolutional network is used to learn an empirical relationship between chemical structures and different semantic descriptors of odor. The information from this relationship (in the form of embeddings) is used as the input for another artificial intelligence model, which is trained to make predictions of odor threshold values from chemical structures. This methodology is named transfer learning and is the main underlying principle behind the prediction model construction.

The predictive performance of the developed model will then be compared to a different prediction model that uses generic descriptors of chemical structures (Extended Connection Fingerprint 4). This is done to evaluate how efficient the transfer learning is in this context. If the prediction using the generic descriptor is better, then the transfer learning is not justified. In contrast, the efficiency of this technique is demonstrated.

**Fig. 1:**
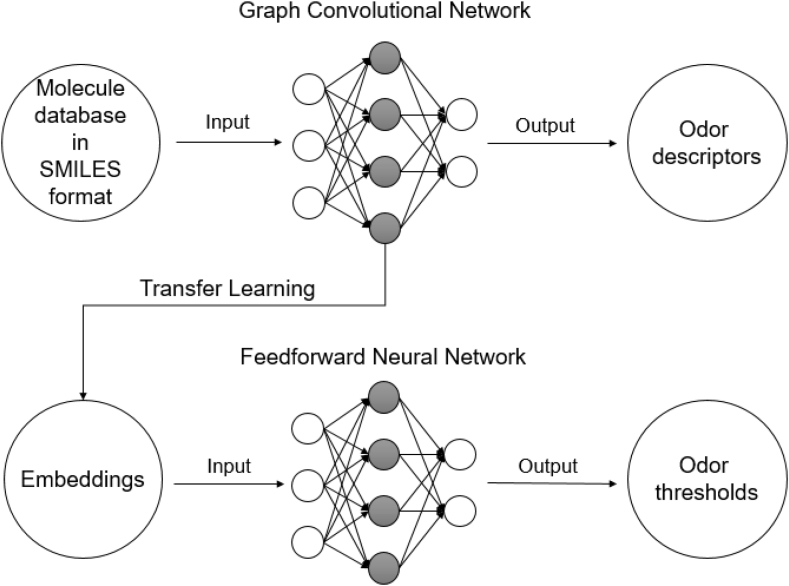
Diagram of the proposed methodology.

Fig. 1 shows a diagram summing up the described methodology.

### 2.1 SMILES

This section will describe SMILES (simplified molecular-input line-entry system) notation. That is a line notation in which the chemical structures were converted to be processed by the models developed in this work. As seen in Fig. 1, the conversion to SMILES notation is the first step of the proposed strategy.

SMILES is a chemical notation language that David Weininger introduced in the 1980s [9]. At that time, the language notations were effective for their intended purpose, but the rules of those notations became too cumbersome to deal with as the structures of molecules increased in complexity. SMILES is more accessible to chemists while retaining enough flexibility and can be more efficiently used with modern computer systems. However, a formal specification of SMILES was never published, which resulted in ambiguities that led to differences in implementation. Thus, in 2007, eMolecules, Omc. Developed OpenSMILES. An open standard for the SMILES language, which addresses several shortcomings of the unpublished formal specifications, that had been developed up to that point. This implementation is used in RDKit, the cheminformatics toolkit used in this work.

SMILES notation was used in order to represent chemical structures so they can be processed by the neural network models used throughout this work. This notation allows for molecular structures to be converted to two dimensional representations, like graphs, as required by the first neural network used in this work, a graph convolutional network.

The transcription of molecular structures to SMILES format requires adherence to a set of simple rules, with a subset of 4 being outlined by Weininger (1988) [9] as sufficient for the transcription of most organic compounds.

1. Atoms are represented by atomic symbols;
2. Double and triple bonds are represented by = and #, respectively;
3. Branching is indicated by parenthesis;
4. Ring closures are indicated by matching digits appended to symbols.

**Fig. 2:**
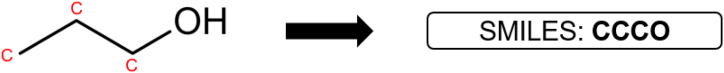
SMILES conversion example.

A simple example of conversion to SMILES format is seen in Fig. 2.

### 2.2 Graph convolutional networks

Graph Convolutional Networks (GCN) were used to build this work's first neural network model, where odor descriptors of chemical compounds are predicted, as seen in Fig. 1. This type of network has been shown to provide a good representation of the underlying empirical relationship between chemical structures and odor [10]. The network will process the graphs obtained from the chemical structures in the SMILES format, and the embeddings from the network will be extracted and used in the following network.

Convolutional neural networks (CNNs) are a type of neural network that specializes in processing data that has a grid-like topology and employs convolution, a mathematical operation on two functions of a real-valued argument that expresses how the shape of one is modified by the other [11]. CNNs are some of the most commonly used ANNs, finding important real-life applications such as image classification [12,13], object detection [14,15] and speech recognition [16,17]. In this work, they will be adapted to be employed in the presented context.

Courville et al. (2016) [11] pointed out that convolution employs three ideas that may improve machine-learning systems and that can motivate the use of convolution in neural networks in the context of this work.

• Sparse interactions – CNNs do not have every input unit interacting with every output unit. This leads to decreased parameter storage, reducing computational requirements for the model and making them less prone to overfitting;
• Parameter sharing – in a traditional neural network, the weight matrix elements are only applied once, multiplying them by an element of the input matrix. In CNNs, the weight matrix elements are tied to other elements applied elsewhere. This reduces the number of weights in the network, leading to reduced training time due to a decreased number of weight updates during training;
• Equivariant translation – the aforementioned parameter-sharing property causes the convolutional layer to have equivariance to translation, meaning that if the input of a function changes, the output changes in the same way.

Graphs can be naturally observed in several types of applications, such as social analysis, computer science and biology. The properties of graphs allow the capturing of structural relations among data, which is more advantageous than analyzing data by itself [18]. The complex nature of the structure of graphs can often be an obstacle to understanding the information that can be obtained from their analysis. Graph-structured data is non-Euclidean in nature, and one potential way of dealing with this complexity is to learn the representation of graphs in a low-dimension Euclidean space through embedding techniques, including both traditional graph embedding methods, as well as network embedding methods. This is accomplished in a way where the essential graph properties can be preserved.

The traditional methods have seen success in these applications, but they can be quite limited in their learning mechanisms, carrying the possibility of failing to uncover the more complex patterns behind the graph structures. On the other hand, deep learning models are more powerful.

### 2.3 GCN training

This section will describe some of the intricacies of the training of GCNs to explain where it differs from the more conventional neural network models.

Graph convolution operators in GCNs require propagating embeddings using the interaction between nodes in the graphs. This significantly complicates the training process. Contrary to other types of neural networks, where the training loss can be perfectly decomposed into individual terms on each sample, the loss term in GCNs depends on many other nodes. Due to this node dependence, the training of GCN's is rather slow and computationally taxing, as backpropagation needs to store all the embeddings in the computational graph in GPU memory [19].

There are several algorithms for GCN training with different characteristics, as well as pros and cons. Three factors are important for evaluating training algorithms: memory requirements, time per epoch and convergence speed per epoch. Two examples of GCN training techniques include.

• Mini-batch stochastic gradient descent [20]: to reduce memory requirements due to each update only being based on a mini-batch gradient. Fast convergence can sometimes be difficult to attain due to the neighborhood expansion problem;
• Cluster-GCN [19]: to address shortcomings of stochastic gradient descent methods, mainly in regards to computational cost. Cluster-GCN samples a block of nodes that associate with a dense subgraph identified by a graph-clustering algorithm and restricts the neighborhood search within this subgraph for each step. According to the authors, this methodology leads to improved memory and computational efficiency, with similar test accuracy as the other training methods.

### 2.4 Database building and transfer learning

The first step was to build a database for establishing an empirical relationship between molecular structures and semantic descriptors of odor. According to available perfume industry data, this was accomplished by associating each molecule present in the database with a number of semantic odor descriptors. A database of 273 molecules and their respective descriptors was created (a sample of this database can be consulted in the supporting information). This database contains the molecules’ respective SMILES entries and semantic odor descriptors. This information was scraped from chemical property databases and a perfume ingredient database, respectively. The descriptors used are common odor descriptors such as “fruity” and “floral”. The aforementioned Table S1 of the supporting information includes the semantic descriptors for the molecules present in the sample. This database was used to perform the learning transfer by training a graph convolutional network, as mentioned in the last section, that was created to predict the semantic descriptors.

In order to develop a neural network for predicting odor thresholds, building a suitable database to be used for training the model is essential. For this work, a database of chemical compounds expressed in SMILES notation paired with their respective detection odor thresholds needed to be built. The odor threshold values were obtained from the 2nd edition of Odor Thresholds for Chemicals with Established Occupational Health Standards [3], a database reference of chemical odor thresholds that contains data for 295 molecules. The odor threshold data used in this study has been sourced from authentic sensory tests from the literature. These tests involved human subjects in controlled settings to determine the perception thresholds of various odors. Such sensory data offers a unique and invaluable perspective as it is grounded in actual human experience and perception, providing a robust foundation for the proposed model.

This database was checked against a perfume industry database in order to create a new database consisting exclusively of compounds used in the perfume industry. This led to the creation of a database that included 45 molecules (the database in its entirety can be consulted in the supporting information), with their respective SMILES notation and odor detection thresholds.

In many cases, the database listed several values for the detection of odor thresholds, with each one generally originating from a different piece of research. The lowest odor detection threshold was used for this work in molecules having multiple values. The authors decided to take this approach because the lowest value represents the lowest known measurement of a certain threshold, according to the used reference database. The lowest known value was determined to be the safest option for the scope of this work and its potential future implementations.

The newly created database is then used in conjunction with a graph convolutional network that was created to establish an empirical relationship between molecular structures and semantic descriptors of odor. To help mitigate the potential shortcomings brought on by the use of a relatively small dataset (45 structures), the concept of transfer learning will be used. Transfer learning is taking knowledge acquired from a previously solved task and applying it to a different, closely related task [21].

**Fig. 3:**
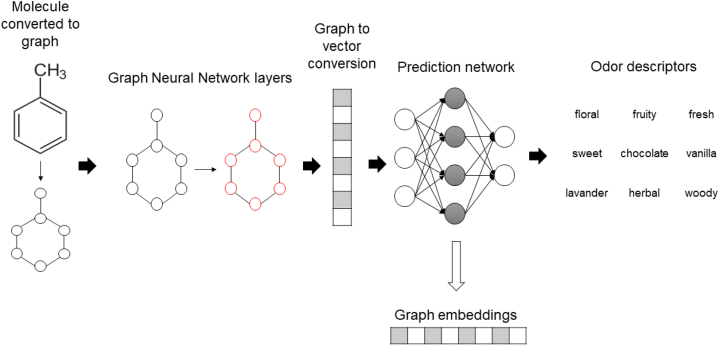
Methodology for the transfer learning process.

In this case, the embeddings from the odor descriptors prediction network will be extracted and used as inputs for the odor threshold prediction network. This resulted in a dataset wherein each structure has a corresponding vector of 128 numbers. It is assumed that using transfer learning will result in a more accurate model due to the inclusion of information from a different network. It can be understood that embeddings represent the empirical relationship between molecular structures and semantic odor descriptors. This relationship is assumed to be very similar to molecular structures and odor thresholds, with the underlying assumption that the physical and chemical mechanisms behind both relationships are similar. The methodology described in this section is summarized in Fig. 3.

### 2.5 Benchmark network

A different odor prediction neural network was also developed in parallel, based directly on molecular structures without resorting to transfer learning – an approach henceforth referred to as generic descriptors, in order to distinguish it from the other model, based on specific descriptors. For each molecule in the dataset, its SMILES notation was converted to its Morgan fingerprint (ECFP4) [22], which is a representation of a molecular structure as a numerical vector – making it viable to be processed by a neural network model. The Extended Connection Fingerprint 4, ECFP4, was derived from the Morgan algorithm, originally developed in 1965 [22] to compare two molecules with different atom numberings and determine if they are identical. The Morgan algorithm assigns a numerical identifier to each atom in the molecule by using a rule that encodes the numbering invariant atom information into an initial atom identifier and, afterwards, by using the identifier from the previous iteration [23].

The ECFP algorithm makes the following changes to the Morgan algorithm.

• ECFP generation terminates after a predetermined number of iterations rather than after identifier uniqueness is achieved;
• Use of optimization algorithms, which is possible because the iteration process does not need to be carried out to completion. Instead, a set number of iterations are carried out.

ECFP4 was chosen as the generic molecular descriptor for this work, as it has a simple to use implementation on RDKit and will be used to develop a predictive model based on generic molecular descriptors, whose performance will be compared to the model based approach proposed in this work.

A feedforward neural network (FNN) was used as a benchmark instead of a GCN in order to provide a closer comparison with the transfer learning approach, which is also based on a FNN. Furthermore, during the development of this work, GCNs were tested as benchmark networks, and the results of that test were shown to be extremely poor, casting serious doubts on the potential of GCNs to be used as benchmark networks.

The performances of both networks were compared, using the mean squared errors of the predictions (in relation to the values of the test dataset), in order to ascertain if the transfer learning approach would lead to a more accurate prediction model.

Furthermore, the overall performances were compared to the arithmetic mean of the datapoints in the test dataset. Hence, providing a baseline for the comparison, since this would be the simplest approach to prediction in the absence of any other model.

### 2.6 Cross-validation approach

Odor thresholds are very difficult to measure, involving a complex process that requires the usage of human subjects. For this reason, data on odor thresholds is very limited and thus, performing a validation of the model with a large number of components is not feasible.

Established techniques in the literature facilitate statistical validation for neural network models, even with limited data. We employed a model validation technique inspired by Schenker et al. (1996) [24] proposed a cross-validation model for neural network models with limited data], which has been shown to be effective even for datasets smaller than ours. By leveraging this cross-validation method, we aimed to ensure the statistical validity of our models. A cross-validation approach based on the one presented by Schenker et al. was employed in this study.

The procedure for this approach is as follows.

• The database of 45 molecules was randomly split in a proportion of 50 %–50 % in order to create two distinct data sets, training sets A and B (with 22 and 23 molecules each, respectively);
• Two different neural network models were trained: model A and model B;
• Model A was trained with training set A and Model B was trained with training set B. The training sets were split 60 %–40 % between training and validation;
• Model A was tested with the entire training data from training set B (23 molecules) and Model B was tested with the entire training data from training set A (22 molecules).

This procedure was repeated for both the generic descriptor and specific descriptor networks. Then, the average mean squared error for both test performances of each descriptor were determined and are displayed in Table 12.

## 3 Results and discussion

### 3.1 GCN training

The database was randomly split into 60 % training data, 20 % validation data and 20 % test data.

A mini-batch gradient descent method was used in the training process. A AUC-PRC (area under curve – precision recall curve) method and F1 score were used as metrics to evaluate the performance of GCN model.

The structure for the GCN model consisted of 3 graph convolutional layers and 2 linear layers. The optimization of the network hyperparameters was carried out by the ASHA algorithm [25]. These hyperparameters are shown in Table 1.

**Table 1:** GCN model hyperparameters.

Number of neurons (GCN layer 1)	64
**Number of neurons (GCN layer 2)**	128
**Number of neurons (GCN layer 3)**	64
**Number of neurons (Linear layer 1)**	128
**Number of neurons (Linear layer 2)**	64
**Learning rate**	0.0013
**Dropout rate 1**	0.5
**Dropout rate 2**	0.5
**Batch size**	16

After the hyperparameter search, the network was retrained with the optimal structure for 300 epochs (using early stopping with a patience of 30 epochs), resulting in the training and validation optimal values for the AUC-PRC metric shown in Table 2.

**Table 2:** AUC-PRC optimal values for training and validation of GCN model.

Training AUC-PRC	0.3121
Validation AUC-PRC	0.2399

**Fig. 4:**
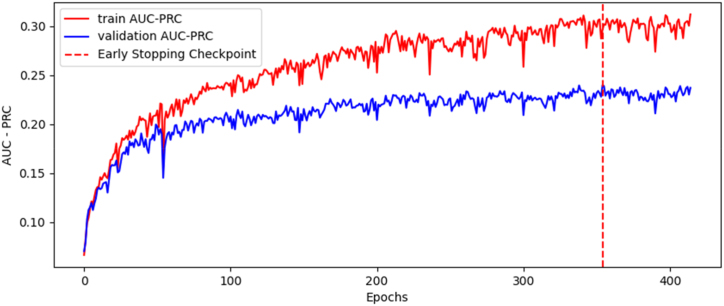
Evolution of AUC-PRC value during retraining.

The training process can be represented by the AUC-PRC progression, as seen in Fig. 4.

The optimal training and validation values for F1 are listed in Table 3.

**Table 3:** F1 optimal values for training and validation of GCN model.

Training F1	0.06281
Validation F1	0.04835

**Fig. 5:**
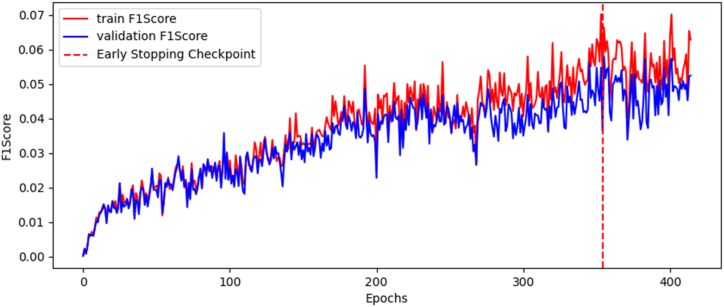
Evolution of F1 value during retraining.

Fig. 5 shows the progression of F1 for the training process.

Although our current analysis indicates a low F1 score, this is not necessarily a cause for concern in this context due to the high AUC-PRC (Area Under the Precision-Recall Curve). The F1 score is a metric often used in the evaluation of binary classification tasks - tasks that involve distinguishing between two classes. It's a harmonic mean of precision and recall, two other essential metrics in classification problems. The F1 score combines these two metrics, balancing them by giving equal weight to precision and recall. However, this is precisely where its limitations can become evident. By giving equal importance to precision and recall, the F1 score assumes that it's just as costly to miss a positive instance (a problem of recall) as it is to incorrectly predict a positive instance (a problem of precision). On the other hand, the AUC-PRC is a performance measure that uses Precision-Recall curves, which plots precision against recall at different threshold settings. Unlike the F1 score, which gives a single-point estimate, the AUC-PRC provides an aggregate performance measure across all possible classification thresholds. In this scenario, we have a dataset where the cost of false positives and false negatives are considerably different. Thus, the AUC-PRC is often more informative than the F1 score.

### 3.2 Feedforward neural network (FNN) training: specific descriptor based ANN

Following the cross-validation strategy described in the methodology, the database of 45 molecules was used to generate two different training sets A and B of 22 and 23 molecules, respectively, that were used to train two ANN models, A and B. Then the entire training data from a network was used as a test dataset for the other network and vice-versa. The splitting process was randomized.

To prevent the model from producing negative numbers as predictions, which would represent a physical impossibility, both training sets were converted to their natural logarithms. These converted sets would be fed to the model, and the predictions would then be “converted back” by applying the exponential function to the predictions so that they can be directly compared to the test datasets.

The training data shown in this section refers to Model B as an illustrative example. This example was chosen because it resulted in the best performance when compared to the baseline criteria described in section 2.5.

The first step to build the prediction neural network model is to generate a hypermodel whose hyperparameters will be selected by an optimization algorithm, the Hyperband algorithm. The search limits given to the Hyperband were set as presented in Table 4, and the values of the optimization parameters are given in Table 5.

**Table 4:** Search limits used with the Hyperband algorithm.

	Lower limit	Upper limit
Number of hidden layers	1	3
Number of neurons per hidden layer (increment of 32)	96	512

**Table 5:** Optimization algorithm parameters.

Optimizer algorithm	Adam optimization algorithm		
Learning rate	1.0 × 10^−3^	1.0 × 10^−4^	1.0 × 10^−5^

The parameters used for the Hyperband algorithm itself are presented on Table 6.

**Table 6:** Hyperband algorithm parameters.

Maximum number of epochs to train one model	100
Reduction factor for the number of epochs and number of models for each bracket	3

The algorithm was configured to seek the set of hyperparameters that minimized validation loss for the model, the loss calculated on the validation set, which is randomly selected before the optimization process from the initial database (40 % split).

After running the Hyperband algorithm, the parameters for the best-performing model are found in Table 7.

**Table 7:** Parameters for the best-performing model.

Number of hidden layers	3
Number of neurons (1st hidden layer)	480
Number of neurons (2nd hidden layer)	352
Number of neurons (3rd hidden layer)	416
Learning rate for Adam algorithm	1.0 × 10^−3^
Minimum validation loss (MSE)	24.23

The number of parameters for each layer are listed in Table 8.

**Table 8:** Parameters for each layer of the model.

Layer (type)	Number of parameters
1st Hidden Layer	61920
2nd Hidden Layer	169312
3rd Hidden Layer	146848
Output Layer	417

**Fig. 6:**
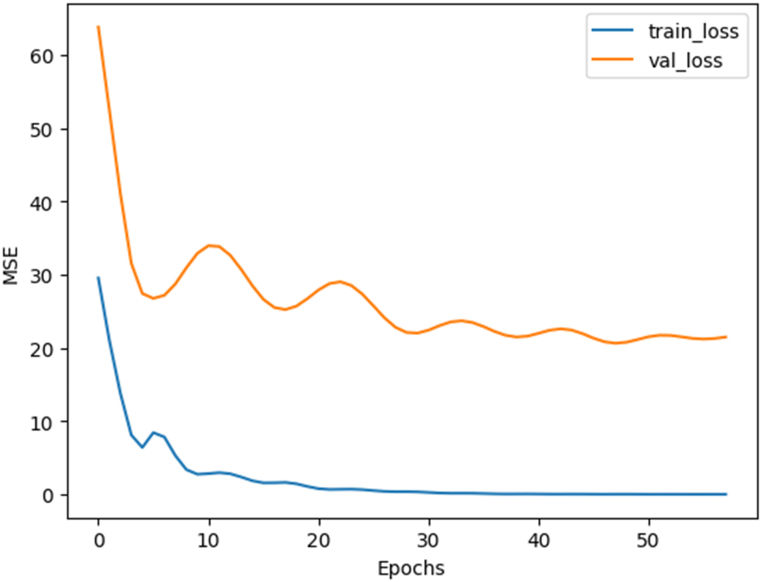
Evolution of training and validation losses for the specific descriptor ANN.

The next step is the actual training process. The training was performed for 150 epochs. Early stopping was used in this training, with a patience value of 10 (number of epochs without improvement on validation error before training is stopped). The evolution of training and validation losses is represented in Fig. 6.

### 3.3 Benchmark ANN training: generic descriptor based ANN

In order to compare the methodology proposed in this work, an ANN was identified by using a generic descriptor, the Morgan descriptor solely, as described in the methodology. Once again, the training data shown in this section refers to Model B, as an illustrative example. Model B of the benchmark networks was selected in order to provide a direct comparison of test performances in Table 11. The hyperparameters search space for this neural network model were identical to those listed in Table 4, Table 5, Table 6. The parameters for the generated model are listed in Table 9.

**Table 9:** Parameters for the benchmark model.

Number of hidden layers	3
Number of neurons (1st hidden layer)	448
Number of neurons (2nd hidden layer)	512
Number of neurons (3rd hidden layer)	416
Learning rate for Adam algorithm	1.0 × 10^−3^
Minimum validation loss (MSE)	15.40

The number of parameters for each layer are listed in Table 10.

**Table 10:** Parameters for each layer of the benchmark model.

Layer (type)	Number of parameters
1st Hidden Layer	459200
2nd Hidden Layer	229888
3rd Hidden Layer	213408
Output Layer	417

**Fig. 7:**
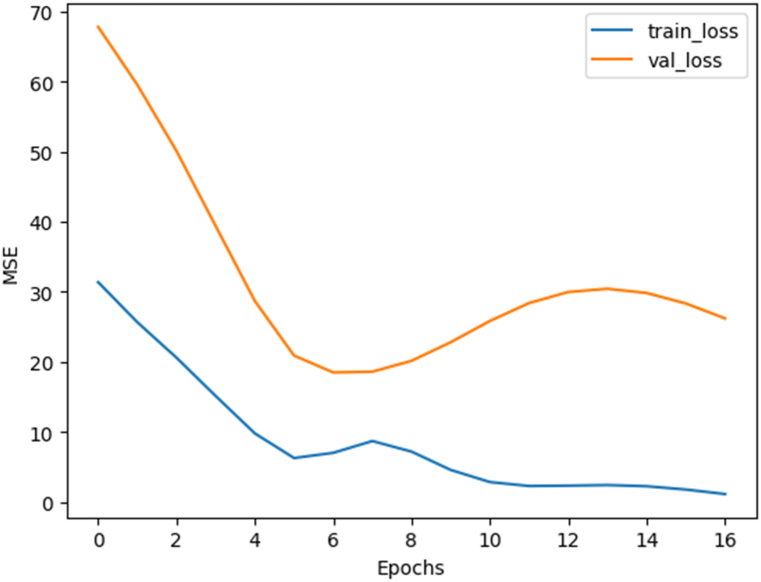
Evolution of training and validation losses for the generic descriptor ANN.

Like in the previous model, training was performed for 150 epochs. Early stopping was enabled with a patience value of 10. The evolution of training and validation losses is shown in Fig. 7.

### 3.4 Predictive performance comparison

Finally, after having all the ANN models identified, it was possible to compare the performance of each strategy in predicting the ODT and evaluate the performance of the proposed method. This was done by using the squared errors for the predictions by both networks. Furthermore, a “dummy regressor” was used to benchmark the ANN approaches. Hypothetically, in the absence of any prediction model, the best model should be the arithmetic average of the available data. If the ANN models are better than the average, this means that they are the best source of prediction available. Thus, the arithmetic mean is used as a baseline comparison. The arithmetic mean was computed from the database. In order to provide an example of a test performance, the mean squared errors of the approaches for predicting the test data are compared in Table 11, along with the individual ODT predictions for the 22 chemical species from the test dataset. This test data is the training data used to train the other network obtained from the cross-validation test (training set A). The final row of Table 11 lists the mean squared errors for the ODT prediction approaches.

**Table 11:** ODT predictions and the mean squared errors for all the prediction models.

Component	SMILES notation	ODT (ppm) [3]	Benchmark model (ppm)	Transfer learning model (ppm)
Mesityl Oxide	O=C(C)C=C(C)C	0.017	0.00756	0.0804
Butyraldehyde	CCCC=O	0.0003	0.00392	0.0171
Isobutyl Acetate	C(C) (=O)OCC(C)C	0.008	0.00109	0.157
alpha-Pinene	CC1=CCC2CC1C2(C)C	0.00006	0.00194	0.0238
2-Methyl Butyl Acetate	CCC(C)COC(=O)C	0.026	0.000511	0.0192
Vanillin	COC1=C(C=CC(=C1)C=O)O	1.6 × 10^−7^	0.00102	0.00651
Camphor	CC1(C2CCC1(C(=O)C2)C)C	0.0026	0.00338	0.0513
*sec*-Butyl Acetate	CCC(C)OC(=O)C	0.0025	0.000704	0.187
n-Amyl Acetate	CCCCCOC(C)=O	0.007	9.48 × 10^−5^	7.31 × 10^−5^
Methyl Isobutyl Ketone	C(C(C)C)C(=O)C	0.03	0.00321	0.137
n-Valeraldehyde	CCCCC=O	0.0004	0.00130	0.00645
1-Hexanol	CCCCCCO	0.0024	0.000369	0.00357
Naphthalene	C1=CC=CC2=CC=CC=C12	0.0019	0.00979	0.00292
Diisobutyl Ketone	C(C(C)C)C(=O)CC(C)C	0.103	0.00491	0.0998
o-Cresol	CC1=CC=CC=C1O	5 × 10^−5^	0.00212	0.00228
2-Methylnaphthalene	CC1=CC2=CC=CC=C2C=C1	0.00069	0.000806	0.00274
n-Butyl Alcohol	CCCCO	0.0033	0.00279	0.00352
1-Pentanol	CCCCCO	0.0055	0.00145	0.00251
Acetophenone	CC(=O)C1=CC=CC=C1	0.00024	0.000770	0.00197
*tert*-Butyl Acetate	CC(=O)OC(C) (C)C	0.008	0.00346	0.0361
Diethyl Ketone	C(C)C(=O)CC	0.85	0.00850	0.222
Furfural	C(C1=CC=CO1)=O	0.002	0.00202	0.0258
**Mean Squared Error**	–	–	0.0327	0.0214

The arithmetic mean of the test set was 0.0487. The mean squared error for the predictions of this mean was 0.0310, meaning that the specific descriptor model showed better performance than the baseline in this particular test. However, this was not the case for the generic descriptor model.

Table 12 shows the average mean squared errors for the test performances of each model trained as a result of the cross-validation process. The arithmetic mean of the test data for each case is also shown.

**Table 12:** Average mean squared errors of the test performances for the cross-validation approach.

	Model A	Model B	Average MSE
Benchmark model	0.0113	0.0327	0.0220
Transfer learning model	0.0144	0.0214	0.0179
Arithmetic mean	0.0125	0.0310	0.0218

The average mean squared error for the specific descriptor network is lower than the generic descriptor network and the arithmetic mean. This outcome provides some support to this work's main hypothesis: it may be possible to transfer learning from a related prediction problem to obtain more accurate prediction results when compared to a network based solely on molecular structures. However, in this study, the improvements in performance could also be attributed to a change of input characteristics.

These results could suggest that the model proposed in this work is a viable prediction model and one that can potentially be developed further and implemented into future works that require the knowledge of odor threshold values, thus mitigating the potential problem of the unavailability of required odor threshold value data.

## 4 Conclusions

The goal of this work was the creation of a machine learning prediction tool for odor thresholds, an important parameter for the perfume industry. The proposed methodology was expressly designed with the challenges faced by real-life industrialists in mind. While the perfume industry might possess a more extensive dataset on ODT, the proprietary nature and the significant costs associated with this data mean it is rarely made public. Despite the constraints of a small database in our study, the methodology outperformed existing prediction methods.

In order to achieve the aforementioned goal, the concept of transfer learning served as the basis for the design of the machine learning models used in this work. In other words, information obtained from a problem was applied in a different, albeit similar, problem. In this case, we used the embeddings from a graph convolutional network that establishes an empirical relationship between semantic descriptors of odor from molecular structures of a database of chemical compounds, as inputs in a feedforward neural network used to predict odor thresholds. As mentioned earlier, there is an assumption of an underlying correlation between molecular structures and odor thresholds. Through the proposed methodology, it may be possible to leverage this correlation without having to rely on threshold data. The performance of the threshold prediction network was compared to a benchmark network developed simultaneously, which used molecular structures without the use of transfer learning in the form of Morgan molecular fingerprints.

This prediction model is not without its limitations, as it was designed to work under limited training data, which creates doubts about the ability to improve its predictive performance in a situation where there is a larger selection of data available. Moreover, the range of chemical species that can be tested with this model can be limited, as there must exist information about their odor descriptors for the model to be able to predict their ODTs.

The results suggest that our proposed model performs better than the benchmark and baseline models, suggesting that the strategy of transfer learning may provide an edge in odor threshold prediction when compared to a model based on a direct relationship between molecular structures and odor thresholds. However, the change of input type could have also led to the changes in performance. Therefore, this study may provide some evidence that using transfer learning could be a viable technique in odor threshold prediction, with potential for further improvements in potential future works.

## Declaration of competing interest

The authors declare that they have no known competing financial interests or personal relationships that could have appeared to influence the work reported in this paper.
