# Supplementary material for: A framework for predicting odor threshold values of perfumes by scientific machine learning and transfer learning
Source: Heliyon. 2023 Oct 10;9(10):e20813. doi: 10.1016/j.heliyon.2023.e20813 (PMC10589844; doi:10.1016/j.heliyon.2023.e20813)
Supplement: Multimedia component 1 [file mmc1.docx]

**Supplementary Material**

**A framework for predicting perfumes odor threshold value by Scientific Machine Learning and Transfer Learning**

Luis M. C. Oliveira^*^, Vinícius V. Santana, Alírio E. Rodrigues, Ana M. Ribeiro, Idelfonso B. R. Nogueira^*^

**Table S1** – Sample of the original ODT database (20 molecules).

| Name | SMILES | ODT (ppm) | Semantic odor descriptors |
| --- | --- | --- | --- |
| Acetic Acid | CC(O)=O | 0.0004 | sharp, pungent |
| Acetic Anhydride | O=C(OC(=O)C)C | 0.12 | sharp |
| Acetone | CC(=O)C | 0.4 | ethereal, pear |
| Acetophenone | CC(=O)C1=CC=CC=C1 | 0.00024 | pungent, nutty, aromatic, gerard, almond, strawberry, fruity, spice, coumarin, floral, cherry, vanilla |
| Acrolein | O=CC=C | 0.0036 | almond, cherry |
| Allyl Alcohol | C=CCO | 0.51 | mustard, pungent |
| n-Amyl Acetate | CCCCCOC(C)=O | 0.007 | banana, apple, ether, fresh, pear, juicy, fruity |
| Benzaldehyde | C1=CC=C(C=C1)C=O | 0.0015 | powdery, nutty, aromatic, gerard, almond, rose, fruity, bitter, spice, coumarin, sharp, cherry |
| Benzyl Acetate | CC(=O)OCC1=CC=CC=C1 | 0.00016 | apple, fresh, aromatic, gerard, fruity, plum, floral, jasmin, cherry, apricot |
| Biphenyl | c1ccccc1-c2ccccc2 | 0.00052 | pungent, rose, green, geranium |
| n-Butyl Acetate | CCCCOC(=O)C | 0.00013 | gerard, ether, sharp, fruity |
| Citral | O=CC=C(C)CCC=C(C)C | 0.000024 | gerard, green, sharp, lemon, fresh, juicy |
| Cyclohexanol | C1CCCCC1O | 0.058 | camphor, phenol |
| Ethyl Acetate | C(C)(=O)OCC | 0.09 | gerard, ether, grape, musty, weedy, fruity, pineapple |
| Isoamyl Alcohol | C(CC(C)C)O | 0.00169 | cognac, banana, pungent, alcohol, wine, coffee, gerard, fruity, chocolate, cocoa, plum, cherry, beverage, apricot |
| Methyl Acetate | C(C)(=O)OC | 0.17 | cognac, gerard, ether, fruity, wine |
| Methyl n-amyl Ketone | CCCCCC(=O)C | 0.00075 | banana, creamy, coconut, cinnamon, herbal, gerard, fruity, spice, green, lavender, cheese |
| 1-Octanol | CCCCCCCCO | 0.0009 | aldehyde, floral, gerard, orange, green, fatty, rose, coconut, waxy, citrus |
| Piperidine | C1CCNCC1 | 0.14 | floral, animal |
| Vanilin | COC1=C(C=CC(=C1)C=O)O | 1.6×10^-7^ | creamy, gerard, chocolate, caramel, vanilla |

**Table S2** –Final database in its entirety, containing only molecules used in perfumery.

| Name | SMILES | ODT (ppm) |
| --- | --- | --- |
| Acetophenone | CC(=O)C1=CC=CC=C1 | 0.00024 |
| n-Amyl Acetate | CCCCCOC(C)=O | 0.007 |
| Benzaldehyde | C1=CC=C(C=C1)C=O | 0.0015 |
| Benzyl Acetate | CC(=O)OCC1=CC=CC=C1 | 0.00016 |
| n-Butyl Acetate | CCCCOC(=O)C | 0.00013 |
| sec-Butyl Acetate | CCC(C)OC(=O)C | 0.0025 |
| tert-Butyl Acetate | CC(=O)OC(C)(C)C | 0.008 |
| n-Butyl Alcohol | CCCCO | 0.0033 |
| n-Butyl Lactate | CCCCOC(=O)C(C)O | 4.9×10^-9^ |
| Butyraldehyde | CCCC=O | 0.0003 |
| Camphor | CC1(C2CCC1(C(=O)C2)C)C | 0.0026 |
| Citral | O=CC=C(C)CCC=C(C)C | 0.000024 |
| o-Cresol | CC1=CC=CC=C1O | 0.00005 |
| Cyclohexanol | C1CCCCC1O | 0.058 |
| Diethyl Ketone | C(C)C(=O)CC | 0.85 |
| Diisobutyl Ketone | C(C(C)C)C(=O)CC(C)C | 0.103 |
| Dimethyl Disulfide | CSSC | 0.00029 |
| Ethyl Acetate | C(C)(=O)OCC | 0.09 |
| Ethyl Benzene | C(C)C1=CC=CC=C1 | 0.002 |
| Furfural | C(C1=CC=CO1)=O | 0.002 |
| 1-Hexanol | CCCCCCO | 0.0024 |
| Indene | C1C=CC2=CC=CC=C12 | 0.0027 |
| Isoamyl Alcohol | C(CC(C)C)O | 0.00169 |
| Isobutyl Acetate | C(C)(=O)OCC(C)C | 0.008 |
| Isobutyraldehyde | C(C(C)C)=O | 0.00034 |
| Isooctyl Alcohol | CC(C)CCCCCO | 0.0092 |
| d-Limonene | CC1=CCC(CC1)C(=C)C | 0.0018 |
| Mesityl Oxide | O=C(C)C=C(C)C | 0.017 |
| Methyl Acetate | C(C)(=O)OC | 0.17 |
| Methyl n-amyl Ketone | CCCCCC(=O)C | 0.00075 |
| 2-Methyl Butyl Acetate | CCC(C)COC(=O)C | 0.026 |
| 2-Methylcyclohexanone | CC1C(CCCC1)=O | 0.181 |
| Methyl Isoamyl Ketone | C(CC(C)C)C(=O)C | 0.0021 |
| Methyl Isobutyl Ketone | C(C(C)C)C(=O)C | 0.03 |
| Methyl Isopropyl Ketone | C(C)(C)C(=O)C | 0.51 |
| 2-Methylnaphthalene | CC1=CC2=CC=CC=C2C=C1 | 0.00069 |
| Methyl Propyl Ketone | CCCC(=O)C | 0.028 |
| Naphthalene | C1=CC=CC2=CC=CC=C12 | 0.0019 |
| 1-Octanol | CCCCCCCCO | 0.0009 |
| 1-Pentanol | CCCCCO | 0.0055 |
| Piperidine | C1CCNCC1 | 0.14 |
| n-Propyl Acetate | C(C)(=O)OCCC | 0.048 |
| alpha-Pinene | CC1=CCC2CC1C2(C)C | 0.00006 |
| n-Valeraldehyde | CCCCC=O | 0.0004 |
| Vanillin | COC1=C(C=CC(=C1)C=O)O | 1.6×10^-7^ |
